# Predictive Validity of the Body Adiposity Index in Overweight and Obese Adults Using Dual-Energy X-ray Absorptiometry

**DOI:** 10.3390/nu8120737

**Published:** 2016-11-30

**Authors:** Robinson Ramírez-Vélez, Jorge Enrique Correa-Bautista, Katherine González-Ruíz, Andrés Vivas, Antonio García-Hermoso, Hector Reynaldo Triana-Reina

**Affiliations:** 1 Centro de Estudios para la Medición de la Actividad Física «CEMA», Escuela de Medicina y Ciencias de la Salud, Universidad del Rosario, Bogota, DC 111221, Colombia; jorge.correa@urosario.edu.co; 2 Grupo de Ejercicio Físico y Deportes, Vicerrectoria de Investigaciones, Universidad Manuela Beltrán, Bogota, DC 110231, Colombia; katherine.gonzalez@docentes.umb.edu.co (K.G.-R.); jose.vivas@docentes.umb.edu.co (A.V.); 3 Laboratorio de Ciencias de la Actividad Física, el Deporte y la Salud, Universidad de Santiago de Chile, USACH, Santiago 7500618, Chile; antonio.garcia.h@usach.cl; 4 Grupo GICAEDS, Facultad de Cultura Física, Deporte y Recreación, Universidad Santo Tomás, Bogota, DC 110311, Colombia; hectortriana@usantotomas.edu.co

**Keywords:** body composition, validity, prediction, obesity, adults

## Abstract

The body adiposity index (BAI) is a recent anthropometric measure proven to be valid in predicting body fat percentage (BF%) in some populations. However, the results have been inconsistent across populations. This study was designed to verify the validity of BAI in predicting BF% in a sample of overweight/obese adults, using dual-energy X-ray absorptiometry (DEXA) as the reference method. A cross-sectional study was conducted in 48 participants (54% women, mean age 41.0 ± 7.3 years old). DEXA was used as the “gold standard” to determine BF%. Pearson’s correlation coefficient was used to evaluate the association between BAI and BF%, as assessed by DEXA. A paired sample *t*-test was used to test differences in mean BF% obtained with BAI and DEXA methods. To evaluate the concordance between BF% as measured by DEXA and as estimated by BAI, we used Lin’s concordance correlation coefficient and Bland–Altman agreement analysis. The correlation between BF% obtained by DEXA and that estimated by BAI was *r* = 0.844, *p* < 0.001. Paired *t*-test showed a significant mean difference in BF% between methods (BAI = 33.3 ± 6.2 vs. DEXA 39.0 ± 6.1; *p* < 0.001). The bias of the BAI was −6.0 ± 3.0 BF% (95% CI = −12.0 to 1.0), indicating that the BAI method significantly underestimated the BF% compared to the reference method. Lin’s concordance correlation coefficient was considered stronger (*ρc* = 0.923, 95% CI = 0.862 to 0.957). In obese adults, BAI presented low agreement with BF% measured by DEXA; therefore, BAI is not recommended for BF% prediction in this overweight/obese sample studied.

## 1. Introduction

The high prevalence of overweight and obesity has become a public health problem worldwide [1]. Several studies have illustrated a link between the accumulation of body adipose tissue, metabolic adverse events, and elevated high risk of developing insulin resistance, diabetes, dyslipidaemia, and hypertension [2,3,4]. For example, the risk of diabetes increases by seven-fold in obese men and by 12-fold in obese women, compared to individuals with normal weight [5]. In fact, globally, obesity is a bigger health crisis than hunger, and is the leading cause of death and disability around the world, with burdens expected to increase in coming years [1].

To estimate the magnitude of this problem, various methods are used to identify adults at risk for excess adiposity in order to prevent the development of overweight and obesity comorbidities. In this context, the precise measurement of body composition by sophisticated methods—such as magnetic resonance imaging, computed tomography, DEXA, isotopic measurement of body water, whole body plethysmography, bioelectrical impedance analysis, and underwater weighting [6]. These methods are costly, time-consuming, and frequently difficult to access. Other disadvantages of these techniques include lack of real-time feedback [6] and repeatability. 

On the other hand, indirect markers were used to assess adiposity by various anthropometric indicators, such as body mass index (BMI), waist circumference (WC) and waist-to-height ratio (WHtR) [7]. All these indicators are simple, inexpensive, non-invasive, and validated methods to apply in clinical practice and epidemiological studies [8]. A study by Bergman et al. [9] proposed a new method to determine excess adiposity, which has been labelled the body adiposity index (BAI). BAI is calculated from hip circumference and height measures as follows: BAI (percentage body fat, BF%) = (hip circumference [cm]/height [m]^1.5^) − 18. The equation proposed for BAI was developed with data from 1733 Mexican-American participants (675 males, 1058 females), aged 18–67 years, using DEXA as the standard method [5]. The results confirmed BAI as a valid predictor of BF% when compared to data obtained with DEXA. In addition, it has been widely used in hospitals (i.e., patients with non-dialysed chronic kidney disease) [10] and research areas (i.e., patients with severe obesity from Brazil) [11]. Correlation coefficients between parameters of body composition assessed by BAI were highly related to those determined by DEXA or magnetic resonance imaging (r values between 0.62 and 0.96) [1,9,12,13], but several publications have criticized the accuracy of BAI [14,15]. However, depending of the population studied, results have been inconsistent. 

Recently, validation and accuracy studies carried out in Costa Rica [15] and Brazil [16] included 199 college students (mean age, 18.1 ± 2.6 years) and 706 healthy adults (mean age, 37.3 ± 12.1 years), respectively. The results showed that BAI is not recommended for BF% prediction among Latinos [15,16]. Given the risk of over-nutrition in developing countries, it is necessary to measure its prevalence in vulnerable populations such as Latin-American people to identify high-risk groups and develop preventive interventions [7,17]. Currently, there are few global reports on the prevalence of overweight and obesity—in particular for low-to-middle income countries (LMICs) experiencing rapid nutrition transitions, as is found in countries within Latin America or Africa [7]. LMICs, including Colombia, are an environment to assess body composition, because the prevalence of both underweight and overweight individuals is relatively high; furthermore, an obesity gradient that includes developing countries from even the poorest households has been reported [18]. In addition, the distribution of fat—and especially accumulation of body adipose tissue—may also be important in risk assessment, especially among persons with a lower BMI [19,20]. 

Clinicians and researchers are increasingly interested in the assessment of body composition as part of the obesity treatment plan to help inform treatment decisions and optimize patient outcomes [21]. There is a need for simple indicators of adiposity in the Latin American population, and such indexes may be especially useful to clinicians to estimate health risk or intervention effectiveness in obese adults. BAI was developed and validated in non-Caucasian subjects, and might be adequate in populations of Central [15] and South America [11,12]. To our knowledge, no previous study has compared BAI with DEXA in the detailed assessment of body composition for obese adults of the Colombian population [22,23]. 

This study was designed to verify the validity of BAI in predicting BF% in Colombian overweight/obese adults by comparison to DEXA measurements of adiposity as a criterion measure.

## 2. Methods

### 2.1. Participants

A total of 48 volunteers (54% women) between the ages of 30 and 50 years with abdominal obesity; waist circumference ≥90 cm (men), ≥80 cm (women) or excess weight by body mass index ≥25 and ≤35 kg/m^2^, respectively, participated in this study (Table 1). Emerging observational studies using BAI with overweight/obese adults yielded inconclusive results—with some criticizing the accuracy of BAI [15,16,17,18,19,20]. Thus, in light of these discrepancies (and given the high prevalence of excess adiposity in the Colombian population), in order to clarify the validity of BAI in predicting BF% among overweight and obese adults, we will conduct a cross-sectional study to evaluate the validity of BAI in a Latin American population. Eligible subjects (volunteer) for the present study and those interested in improving health and fitness were invited to a pre-test that included an interview in a private health care institution (Clinica Rangel Pereira IPS) and further assessments performed at the Centre of Studies in Physical Activity Measurements (in Spanish, CEMA), School of Medicine and Health Sciences, University of Rosario, Bogotá, Colombia. Pregnant women, individuals with physical disabilities, and individuals who were bedridden at the time of data collection were excluded. The Institutional Ethics Committee approved the study, in accordance with the latest version of the Declaration of Helsinki (UMB No. 01-1802-2013). After reading and signing an informed consent to participate in the study, volunteers were given an appointment for a testing session at the Centre for Studies in Physical Activity Measurements (CEMA). 

### 2.2. Design and Procedure

This was a cross-sectional analytical study where participants were measured only once. A member of the research team followed up with a phone call to screen for eligibility and explain the main requirements of the study. After completing a general information questionnaire, participants were instructed to wear shorts, a t-shirt, and to remove any metal and jewelry from their person. Anthropometric measurements were taken according to the anthropometrics recommendations of the International Society for the Advancement of Kinanthropometry [24]. Data were collected in the morning, in a single meeting, by the same trained and experienced evaluator. The body weight of the subjects was measured when the subjects were in underwear and without shoes, on electronic scales (Tanita^®^ BC544, Tokyo, Japan). The height of the subjects was measured using a mechanical stadiometer platform (Seca^®^ 274, Hamburg, Germany). We calculated BMI (BMI = weight/height^2^) from the height (kg) and weight (m) measurements. Weight status examined included WHO criteria for obesity (BMI ≥ 30 kg/m^2^) and overweight (BMI ≥ 25 kg/m^2^) [25]. The WC (cm) was measured as the narrowest point between the lower costal border and the iliac crest; in cases where it was not evident, it was measured at the midpoint between the last rib and the iliac crest, using a tape measure (Ohaus^®^ 8004-MA, Parsippany, NJ, USA). Hip circumference (cm) was measured at the widest point around the buttocks, with the tape horizontal and parallel to the ground using a tape with 0.1 mm accuracy (Ohaus^®^ 8004-MA, Parsippany, NJ, USA). BF% for each participant was determined by a whole body DEXA scan (Lunar Prodigy Advance; GE-Medical Systems, Madison, WI, USA) and software (enCore 2006, ver. 10.51.006; GE-Company, Madison, WI, USA), according to the manufacturer’s instructions. Scans were performed and analysed by the same trained operator, according to the laboratory standard protocol. The subjects were instructed to assume a supine position with their arms by their sides and palms in a neutral position. To ensure data quality, the equipment was calibrated daily using a known calibration standard following manufacturer instructions (step phantom scan for body composition calibration). The in vivo coefficient of variation assessed in our laboratory for use in adults is 1.2% for BF%. BAI was calculated from hip circumference and height as previously described [9]. In addition to BAI, we also calculated the WHtR. The mean (SD) of the time interval between the BAI and DEXA measurements was 6.8 (2.6) days. 

### 2.3. Statistical Analysis

Statistical analyses were performed using Statistical Package for the Social Sciences software for Windows version 21.0 (IBM Corporation, Chicago, IL, USA). Distributions were determined by Shapiro–Wilk test. We considered *p*-values below 0.05 to be significant. Statistical analysis included a description of the variables (mean and standard deviation) and *t*-test analyses to check for differences of means. The DEXA method was used as the “gold standard” to determine BF%. Independent samples *t*-tests were performed between sexes to determine differences in BF% and anthropometric characteristics (Table 1). The correlation between variables was assessed by Pearson’s correlation coefficient by sex. In addition, for each sex, paired samples *t*-tests were used to test differences in mean BF% obtained with BAI and DEXA methods. Lin’s concordance correlation coefficient was used to assess the reproducibility between BAI and DEXA by sex [26]. Lastly, we used the graphical approach of Bland–Altman to verify the agreement between BAI and DEXA [27,28].

## 3. Results

Descriptive statistics and between-sex comparisons are shown in Table 1. All of the anthropometric variables—except BF% DEXA, BAI and hip circumference—were higher in women than in men (*p* < 0.05). 

Table 2 shows the partial coefficients of correlation between BAI and different anthropometric measures controlled by sex and age. Overall, BAI has the highest coefficient of correlation with BF% by DEXA (*r* = 0.844, *p* < 0.001), weight (*r* = 0.668, *p* < 0.001), and moderate correlation with BMI (*r* = 0.557, *p* < 0.001). However, stratified analyses according to sex showed that among men, significant correlations were found for BMI (*r* = 0.739, *p* < 0.001), WHtR (*r* = 0.639, *p* < 0.001), and BF% by DEXA (*r* = 0.677, *p* < 0.001) with BAI. In women, significant correlations were found for all measurements evaluated. For women and men, the Lin’s concordance correlation coefficient (*ρc*) was stronger, *ρc* = 0.882 (95% CI = 0.783 to 0.947) and *ρc* = 0.803 (95% CI = 0.525 to 0.918), respectively.

Men and women were then divided according to BF% and, as shown in Table 3, BAI underestimated BF% at all levels of adiposity. Significant differences were found in both sexes with BF% greater than 30% (*p* < 0.05). 

**Figure 1:**
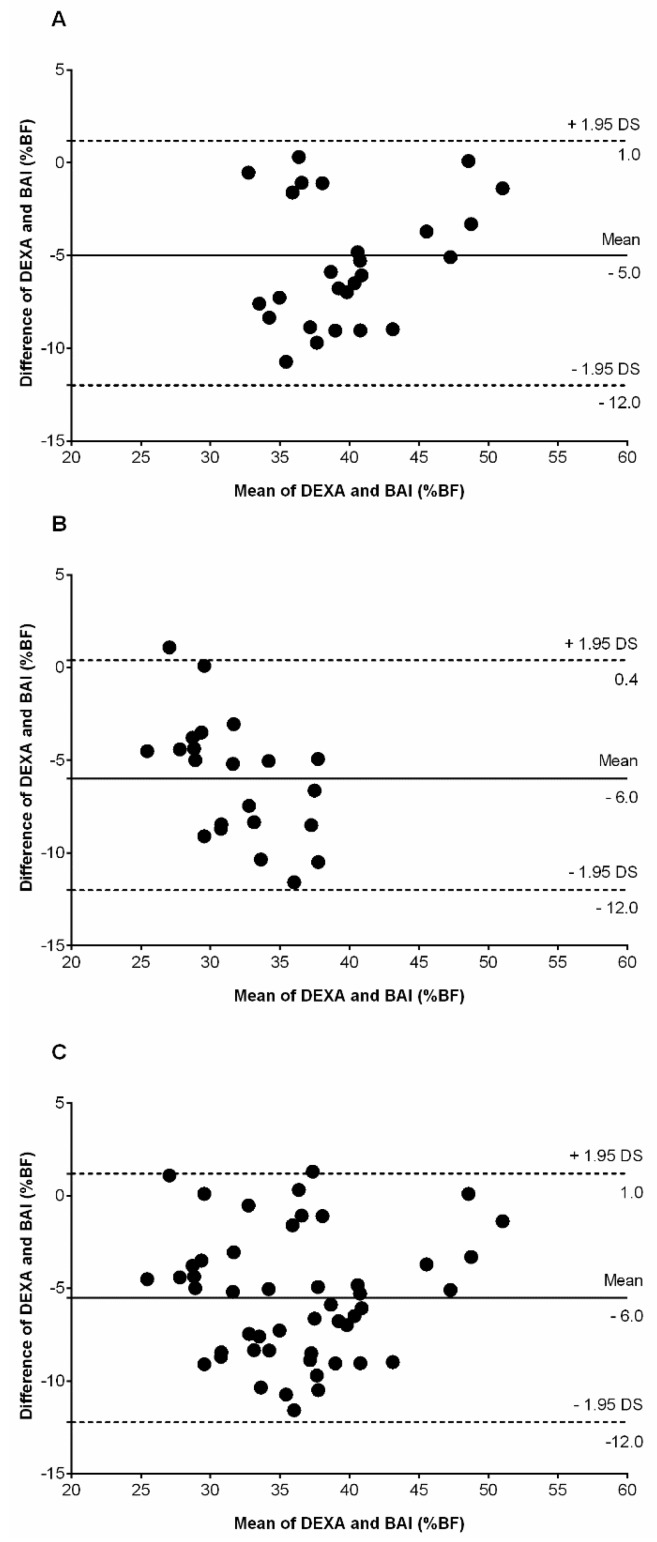
Bland–Altman plots of BF% assessed by DEXA and BAI among (**A**) women; (**B**) men; and (**C**) all participants. The differences between the two methods is plotted against their mean. The solid line represents the mean value from the two methods and dashed lines is SD 1.96.

The Bland–Altman plot (Figure 1) showed BAI underestimating BF% in relation to the “gold standard” in women (A), men (B), and all participants (C). For women, a paired *t*-test showed a significant mean difference in BF% between methods (difference in mean −5.3 (3.3); BAI 37.1 (5.5)% vs. DEXA 42.5 (4.7)%, *p* < 0.001). The bias of the BAI was −5.0 (SD 3.0) BF% (95% CI = −12.0 to 1.0). For men, a paired *t*-test showed a significant mean difference in BF% between methods (difference in mean −6.0 (3.2); BAI 28.8 (3.0)% vs. DEXA 34.8 (4.8)%, *p* < 0.001). The bias of the BAI was −6.0 (SD 3.0) BF% (95% CI = −12.0 to 0.4). For women and men, the bias of the BAI was −6.0 (SD 3.0) BF% (95% CI = −12.0 to 1.0), indicating that the BAI method significantly underestimated the BF% compared to the DEXA method. These plots suggest that differences between the two methods exhibit a regular obvious pattern (proportional bias), with underestimation in both sexes with higher BF%. Indeed, this visual information is verified by the percentage of participants classified as higher level of adiposity and overweight and obesity. 

## 4. Discussion

The purpose of the study was to verify the predictive validity of BAI to estimate BF% in a sample of Colombian overweight and obese adults. The main finding was the lack of predictive validity of BAI for the estimation of BF% compared to DEXA in both sexes. Therefore, BAI is not recommended in this Colombian obese population. The Bland–Altman plots showed a trend of BAI to underestimate adiposity in men and women in relation to the criterion measure DEXA (Figure 1). Another finding was that BAI underestimated BF% in both sexes (bias 6%), mainly at higher degrees of adiposity or weight status. Therefore, the use of BAI does not seem to be a good alternative compared to either waist or hip circumference and BMI.

In this study, BAI and DEXA BF% showed significant high and moderate correlation coefficients in women and men, respectively. However, stratification by sex shows this difference, with DEXA being related similarly among women and among men to BAI, BMI, waist circumference, and hip circumference. For the total population, the correlation between BAI and BF% (measured by using DEXA) (*r* = 0.844; *p* < 0.001) was stronger after adjusting for age and sex. Although differences in the magnitudes of the correlations with DEXA were relatively small, in several instances (e.g., men), the observed correlation with BMI was stronger (*p* < 0.001) than that with BAI.

Although it has been suggested [9,28,29,30,31,32] that BAI can provide an estimate of BF% without the need for further adjustment, our results indicate that these estimates will be systematically biased by sex, level of adiposity, and weight status. On average, BAI underestimated BF% among women and men by 5.0%, biases that are fairly similar to the those reported among 623 European-American adults in the Fels Longitudinal Study [33] and as reported by Freedman et al. [34] among 1151 adults at the Body Composition Unit of the New York Obesity Nutrition Research Center. In both sexes, we found BAI underestimated BF% by about 6%; this may have been due to their high BMIs (mean, 30.7 ± 4.0 kg/m^2^). Because the difference between measures associated with BAI varies substantially by levels of adiposity/weight status and sex (Table 3), it would be expected that sex differences across studies would vary somewhat, depending upon study-specific levels of adiposity. Ethnicity is another factor that greatly influences the shape and body composition of an individual. For example, previous studies in different populations [15,28,29,30,31]—such as European Americans [9,32,33], Mexican Americans [9,34], African Americans [28], and Latin Americans [12,15,16]—which have showed that BAI overestimates BF% at lower levels of adiposity. Similar to our findings, these studies showed that BAI underestimates BF% at higher levels of adiposity (Table 4) [15,31,32,35,36]. 

It was observed that among subjects with percent fat values between 20% and 30%, the highest correlation between BAI and DEXA was observed, as was also the case in other studies [9,16,30]. In a youth population, Thivel et al. [28] found a low association between the BF% estimates determined by BAI versus DEXA. In athletic women, Esco et al. [29] cross-validated BAI with DEXA as the reference method, and found large individual errors when predicting BF%. Furthermore, in a study of overweight and obese postmenopausal Caucasian women conducted by Lemacks et al. [31], BAI underestimated BF% by up to 7.56% compared with DEXA. Chang et al. [30] observed that the BAI method had a tendency to overestimate and underestimate BF% in men and women aged 55–96 years that presented with BF% estimated by DEXA at <15% and ≥40%, respectively. In 102 women (average age 60.3 ± 9.8), BF% by DEXA was significantly lower than that estimated by BAI, with BAI overestimating the BF% by 3.2%. In contrast, Carpio-Rivera et al. [15], in a sample of Costa Rican students, showed that BAI underestimated and overestimated BF% in relation to the “gold standard” in women and men, respectively.

The reasons for this discrepancy are not clear, but as BAI quantifies adiposity based on height adjusted hip circumference, different body fat distributions among populations may be reflected in different BAIs [36]. As for the difference between the sexes, women have higher levels of BF% than men and differences in the distribution pattern of body fat [12], whereas for height, men have higher mean values than women [12]. Differences in the anthropometrics profiles and body composition among ethnic groups can change the relationship between anthropometric measurements and BF%, invalidating the equation in other populations. In addition, weaker associations have been reported between cardiovascular risk factors and BF% by BAI than with WC, WHtR, and BMI [29,36].

Our study has several important limitations. First, our study population may not be entirely representative of the general adult Colombian population, since it only included overweight and obese individuals aged from 30 to 50 years old. Second, our study used DEXA as the reference method of BF% determination. DEXA estimates of BF% have been known to vary between manufacturers and across models. In our case, although the manufacturer of our DEXA (Hologic) was the same as the one used in Bergman et al.’s study [9], the model with its attending scan mode and software was different. Fourth, the extrapolation of an equation for the estimation of BF% based on measurements of body circumference and height for the Colombian population should be viewed with caution, because it is composed of a mixture of Amerindians, Europeans, and Africans, one of the most heterogeneous populations in the world, conferring their peculiar characteristics [12]. 

## 5. Conclusions

Therefore, we conclude that the BAI underestimates DEXA-derived BF% (with a wide 95% limit of agreement) in an overweight and obese sample from Colombia. BAI only provides a modest estimate of BF% in this population. Thus, BAI does not appear to be an appropriate proxy for BF% in overweight and obese Colombian adults; it may be possible that the inclusion of other anthropometric measurements into the BAI equation may provide correction factors to obtain more accurate BF% estimates. We emphasize the importance of a simple and inexpensive method for adiposity estimation in countries where the availability of sophisticated equipment is not wide. Further epidemiological studies examining the utility of BAI for Latin-American populations are still needed for a better understanding of the validity of this new index.

## Figures and Tables

**Table 1:** Characteristics of study subjects as a whole and by sex.

	Women (*n* = 26)	Men (*n* = 22)	Total (*n* = 48)	*p*
Age (years)	42.3 (8.2)	39.3 (6.0)	41.0 (7.3)	0.157
Height (m)	1.63 (7.6)	1.65 (9.0)	1.64 (8.2)	0.375
Weight (kg)	78.2 (12.3)	89.8 (12.4)	83.5 (13.6)	0.002
Waist (cm)	90.7 (8.1)	100.9 (8.8)	95.4 (9.8)	0.001
Hip (cm)	110.3 (9.7)	105.2 (6.2)	107.9 (8.6)	0.038
WHtR	0.57 (0.05)	0.59 (0.04)	0.58 (0.05)	0.272
BF% DEXA	42.6 (4.8)	34.8 (4.8)	39.0 (6.1)	0.001
BF% BAI	37.2 (5.6)	28.8 (3.1)	33.4 (6.2)	0.001
BMI (kg/m^2^)	31.0 (4.5)	30.4 (3.3)	30.7 (4.0)	0.611
BMI ≥ 30 (kg/m^2^) ^a^	13 (50.0)	10 (45.4)	23 (47.8)	0.753

Data are expressed as mean (SD) or ^a^ *n* (%). *p* values are given for comparison between women and men. BAI: body adiposity index; BF%: body fat percentage; DEXA: dual-energy X-ray absorptiometry; WHtR: waist-to-height ratio.

**Table 2:** Partial correlation coefficients between BF% determined by BAI and different anthropometric variables controlled by sex and age.

	Women (*n* = 26)	Men (*n* = 22)	Total (*n* = 48)
BF% DEXA	0.763 *	0.677 *	0.844 *
Weight (kg)	0.696 *	0.465 *	0.668 *
Waist (cm)	0.574 *	0.596 *	0.546 *
Hip (cm)	0.874 *	0.685 *	0.613 *
WHtR	0.667 *	0.639 *	0.336 *
BMI (kg/m^2^)	0.716 *	0.739 *	0.557 *

* All reported correlation coefficients are significant at *p* < 0.001.

**Table 3:** BF% by DEXA and BAI according to different levels of adiposity by sex.

	Women (*n* = 26)					Men (*n* = 22)				
	*n*	BF% by DEXA	BAI	*p* Value	Difference between Measures	*n*	BF% by DEXA	BAI	*p* Value	Difference between Measures
**All**	26	42.5 (4.7)	37.1 (5.5)	0.001	−5.3 (3.3)	22	34.8 (4.8)	28.8 (3.0)	0.001	−6.0 (3.2)
**Level of adiposity (%)**										
20–30	–	–	–	–	–	5	28.8 (1.7)	26.5 (2.3)	0.127	−2.3 (2.6)
31–40	9	37.4 (2.1)	33.1 (3.1)	0.001	−4.2 (1.3)	14	35.3 (3.1)	28.9 (3.0)	0.001	−6.4 (1.2)
>41	17	45.2 (3.2)	39.3 (5.4)	0.005	−5.9 (3.2)	3	42.1 (0.7)	31.9 (1.4)	0.048	−10.2 (1.9)
**Weight status**										
BMI > 25 < 30 (kg/m^2^)	13	39.6 (3.3)	33.7 (2.4)	0.001	–5.8 (3.7)	12	32.4 (4.0)	27.7 (2.9)	0.001	−4.7 (2.8)
BMI ≥ 30 (kg/m^2^)	13	45.5 (4.2)	40.6 (5.8)	0.001	−4.8 (2.9)	10	37.6 (4.2)	30.0 (2.8)	0.001	−7.5 (3.2)

Data are expressed as mean (SD). Level of adiposity (20 to 30; 31 to 40; and >41 BF%) was classified according to the National Health and Nutrition Examination Survey (NHANES) (1999–2004) by DEXA in Spanish population [21].

**Table 4:** Comparison of BAI parameters of the included trials *.

Study	Sample	Age (Years)	Agreement between Measurement Methods/Bias	Main Finding
Present study	22 men, and 26 women	30–50	Bland–Altman plots Women bias 5.0%; Men bias 6.0%	In both sexes, BAI underestimated BF%
Thivel et al. [28]	58 girls, and 61 boys: adolescents	12–16	Bland–Altman plots Bias 3.4%	In both sexes, BAI overestimated BF%
Bergman et al. [9]	1733 Mexican American subjects	20–50	Correlation between DEXA–BAI *ρc* = 0.95	In both sexes, BAI had adequate accuracy
Segheto et al. [16]	331 men, and 395 women	20–59	Bland–Altman plots Women bias 5.0%; Men bias 5.4%	In both sexes, BAI overestimated BF%
Carpio-Rivera et al. [15]	106 men, and 93 women: college students	Mean age 18.9 ± 2.6	Bland–Altman plots Women bias 7.2%; Men bias 2.9%	In women, BAI underestimated BF%; In men, BAI overestimated BF%
Cerqueira et al. [12]	102 womens	Mean age 60.3 ± 9.8	Bland–Altman plots Bias 3.2%	BAI overestimated BF%
Esco et al. [29]	30 women athletes	Mean age 20.0 ± 1.3	Bland–Altman plots Bias 5.8%	BAI overestimated BF%
Chang et al. [30]	483 mens, and 471 womens college students	55–96	Bland–Altman plots Bias 5.1%	BAI overestimated BF%
Lemacks et al. [31]	187 overweight/obese postmenopausal womens	Mean age 55.8 ± 3.3	Concordance correlation coefficient *ρc* = 0.39	Poor agreement strength between DEXA BF% and BAI; BAI overestimated BF%
Vinknes et al. [32]	5193 middle-aged (47–49 years) and elderly (71–74 years) men and womens	47–72	Bland–Altman plots	BAI overestimated adiposity in subjects with lower BF% (particularly in men) and underestimated it in overweight and obese subjects.

* Dual-energy X-ray absorptiometry is cited as a criterion method.

## Abbreviations

The following abbreviations are used in this manuscript

BAI	Body adiposity index
BF%	Body fat percentage
BMI	Body mass index
CEMA	Centre for Studies in Physical Activity Measurements (in Spanish)
DEXA	Dual-energy X-ray absorptiometry
LMICs	Low-to-middle income countries
WC	Waist circumference
WHtR	Waist-to-height ratio
*ρc*	Lin’s concordance correlation coefficient
